# Central optical power of the isolated human lens without zonular tension

**DOI:** 10.1371/journal.pone.0326954

**Published:** 2025-07-01

**Authors:** Ronald A. Schachar, Ira H. Schachar, Shubham Kumar, Farhad Kamangar, Boyd Hunter, Barbara K. Pierscionek, Pamela C. Cosman

**Affiliations:** 1 Schachar LLC, La Jolla, California, United States of America; 2 North Bay Vitreoretinal Consultants, Santa Rosa, California, United States of America; 3 Department of Electrical and Computer Engineering, University of California San Diego, San Diego, California, United States of America; 4 Department of Computer Science and Engineering, University of Texas at Arlington, Arlington, Texas, United States of America; 5 Praxis Optics, Elmira, New York, United States of America; 6 Faculty of Health, Medicine and Social Care, Medical Technology Research Centre Anglia Ruskin University, Chelmsford, United Kingdom; University of Warmia, POLAND

## Abstract

The ability to focus at near is achieved by dynamic changes in the shape of the lens of the eye. The Helmholtz hypothesis of accommodation proposes that, at distance gaze, all of the lenticular supporting zonules are at maximal tension. To bring a near object into focus, this tension is reduced by action of the ciliary muscle. The resultant release of tension allows the elastic lens capsule to mold the lens into a more rounded shape, increasing both its central thickness and central optical power (COP). Based upon Helmholtz’s hypothesis, complete removal of these zonules should result in a rounded shaped lens of maximal COP. Schachar has offered an alternative mechanism of accommodation based upon the distinct actions of the three different groups of lenticular zonules. Schachar believes that for distant objects, all the zonules are under the minimum tension required to maintain lens stability; however, during lenticular accommodation, equatorial zonular tension increases while, simultaneously, the anterior and posterior zonular tension decreases. The selective increase in equatorial zonular tension results from the unique orientation of the different ciliary muscle fiber groups. With this increase in equatorial zonular tension, the peripheral lens surfaces flatten, central surfaces steepen and central lens thickness and COP increase. Schachar’s hypothesis would anticipate that with zonular removal, the COP of the isolated lens would be minimal and diametrically opposite to the high lens COP expected with the Helmholtz hypothesis. In order to determine the COP of the isolated human lens, we obtained, through the kindness of the authors of an independent research study, the x-y coordinates of the central sagittal lens profiles of 10 freshly isolated human lenses (donors aged 20–30 years). These coordinate data were then mathematically utilized by fitting them into Chien, Forbes, Fourier, and elliptical equations. Additionally, the coordinate data was smoothed and fit to third-degree polynomials (S4W 3rd Poly). Independent of which of these equations was employed, within central optical zone diameters of ≤ 3 mm, the COP was found to be minimal. Since the S4W 3rd Poly provided the best fit, it was used to represent lens surfaces in optically modeled eyes. In all modeled eyes, Zernike spherical aberration (SA) coefficients were positive. These findings are consistent with *in vivo* measurements of SA obtained from human eyes while viewing distant visual objects. Having thus demonstrated that freshly removed human lenses, free of zonular tension, have their least COP, it is likely that this condition mimics the physiologic status of the human lens in the eye while attending to the most distant visual objects. In an independent, companion paper, we observed, using interferometric measurements of surface radius of curvatures of 12 fresh, isolated human lenses, obtained from donors aged 20–30 years, that the minimal COP was also associated with the unaccommodated state *in vivo.*

## Introduction

Internally, the eye has two major muscular structures, the iris and the ciliary muscles. The iris constricts in response to light to reduce excess retinal illumination and to optically increase the depth of image focus. The ciliary muscle provides the force to indirectly change tension on the lens capsule and, thereby, alter the shape of the lens. The ciliary muscle has longitudinal, radial and circular muscle fiber groups which are identified by their orientation. The ciliary muscle transmits its force to collagen fibers, that attach to the ciliary body epithelium where the zonules originate. The zonules are named by the locations to which they ultimately insert on the lens capsule. The equatorial zonules originate in the valleys of the ciliary processes of the pars plicata and insert into the equator of the lens capsule. The anterior and posterior zonules originate in the pars plana and insert anterior and posterior to the lens capsule equator.

When viewing at a distant object, the ciliary muscle of the eye is at rest while the zonules provide enough tension to maintain stability of the lens. According to the Helmholtz theory of accommodation, at distance gaze, all of the lenticular supporting zonules are at maximal tension. To bring a near object into focus, this tension is reduced by action of the ciliary muscle. The resultant release of tension allows the elastic lens capsule to mold the lens into a more rounded shape, increasing both its central thickness and central optical power (COP). Based upon Helmholtz’s hypothesis, an isolated lens with the complete removal of these zonules should result in a rounded shaped lens with the highest COP. Support for the Helmholtz postulate has been obtained from *in vitro* studies where young isolated human lenses without zonular tension have been described to be fully accommodated, i.e., with maximum COP [1–3]. However, Helmholtz himself, offered some data that questions this conclusion. He clinically examined the right eyes of three females aged 25–30 years with an ophthalmometer and found anterior lens surface radii of curvature (RoCs) values at far vision (unaccommodated) of 11.9 mm, 8.8 mm and 10.4 mm, and stated that two post-mortem lenses with RoCs of 10.2 mm and 8.9 mm “*agrees well with measurements on the living eyes*” [4]. Stadfeldt in 1896 measured, *in vivo*, the anterior RoC of 11 unaccommodated human crystalline lenses using an ophthalmophakometer [5]. He found that the mean central RoC was 10.5 mm. After removal of six of these crystalline lenses, the mean central anterior RoC measured with an ophthalmometer was 11.4 mm [5]. More recently, Schachar, using a topographer, similarly found the mean ± SD RoC of lenses from donors was 10.5 ± 0.6 mm [6]. These measurements of anterior lens surface RoC are consistent with the physiologic status of the human lens in the eye while attending to the most distant visual objects as predicted by schematic eye models [7]. Our study aims to resolve the seemingly contradictory interpretations of prior findings by measuring the COP of freshly isolated human lenses.

The lens is usually accepted to be an oblate spheroid because semi-ellipses (conics) approximately fit the anterior and posterior lens surfaces [8,9]. The minor axes of the semi-ellipses are coincident with the optical axis of the lens. Consequently, when the curvature of the lens is measured using an elliptical fit, its curvature will appear to increase; i.e., RoC decreases, and optical power increases, with distance from the optical axis because of the inherent curvature changes of an ellipse [10]. This decrease in RoC with distance from the optical lens axis has been observed *in vivo*. Using corrected Scheimpflug photography the vertex RoC of the *in vivo* unaccommodated lens is significantly greater than at the 3 mm central optical zone (COZ). For example, the reported age-related regression formula for *in vivo* unaccommodated anterior 3 mm optical zone RoCs is [11]:


3mmCentralOpticalZoneAnteriorLensRoC(mm)=12.9−0.057·age (1)

where, age is in years.

Therefore, the mean anterior lens RoC at a 3 mm COZ for 28–33 years of age would be 11.2 mm, which is less than the mean conicoid corrected “*apical*” anterior lens RoC of 12.0 mm (12.48 mm, 11.43 mm and 12.18 mm) [12] observed by OCT in three unaccommodated subjects aged 28–33 years.

When accommodating under normal lighting conditions, the pupil generally constricts and, for accommodation of ≥6 diopters, the clinically measured pupil has a diameter less than 4 mm [13]. Since the actual pupil diameter at the surface of the lens is 13% smaller [14], the functional COZ of the anterior lens surface is < 3.5 mm when accommodating ≥6 diopters. Moreover, a 3 mm keratometric COZ is generally used to reliably calculate intraocular lens power.

Therefore, to relate the functional RoC of the lens central surfaces and COP of isolated human lenses to *in vivo* lenses, it is important that RoC values at the same functional COZ diameter of 3 mm are compared. Fortunately, *in vivo* central lens RoCs at the 3 mm optical zone have been measured in young subjects [11].

In addition to considering COZ diameter when assessing lens RoC, the fit and smoothness of the curve must be maximized. Conics, including ellipses, do not fit aspheric surfaces like the human lens very well, and aspheric equations can have smoothness problems [15–19]. Therefore, different approaches are required. Furthermore, to relate *in vitro* to *in vivo* lens topographic measurements, fresh isolated lenses must be measured. Over time, even in preservative media, the isolated lens imbibes fluid causing the anterior surface of the lens to steepen and central lens thickness to increase [6]. In the present study, multiple equations were evaluated for fit, smoothness and COP versus optical zone size to assess the COP of the isolated lens without zonular tension. In the companion paper, vertex RoCs of fresh isolated human lenses from young donors were interferometrically measured objectively and automatically.

## Materials and methods

### Isolated lens profiles

For a previously published study by Mohamed et al. [1], sagittal profile x–y coordinates of fresh, isolated human lenses were obtained using a miniature digital shadow-photogrammetric system at a mean postmortem interval of 37.2 hours. Following written informed consent, these lenses were procured by the Ramayamma International Eye Bank, L.V. Prasad Eye Institute (Hyderabad, India) in accordance with the tenets of the Declaration of Helsinki for research involving human tissue. In addition, the study obtained approval from the Institutional Ethics Committee [1]. For the present study, profilometer centered and aligned x-y coordinates of 10 lenses of unidentifiable donors aged 20–30 years from Mohamed’s et al. study were generously provided by R.C. Augusteyn and A. Mohamed on 27 June 2022 (profilometer centered and aligned x-y coordinate data given in the S1 Table).

### Equations

The RoCs and COPs of 10 isolated lenses were evaluated using the following equations, Chien [19], Forbes [18,20,21], Fourier [2], and ellipse [19]:


$$Chien\;equation:\;y\left(\theta \right)=\;\left(b_{0}+b_{1}\theta^{2}+b_{3}\theta^{4}\right)cos\left(\theta \right)$$ (2)

$$Forbes:\;z\left(\rho \right)=\frac{c\rho^{2}}{1+\sqrt{1-\left(1+K\right)c^{2}\rho^{2}}}+\frac{u^{2}\left(1-u^{2}\right)}{\sqrt{1-c^{2}\rho_{\max}^{2}u^{2}\;}}\sum_{m=0}^{M}a_{m}Q_{m}\left(u^{2}\right)$$ (3)

$$Fourier\;equation:\;\rho \left(\theta \right)=\sum_{n=0}^{10}\left(b_{n}\right)cos\left(n\theta \right)$$ (4)

$$Ellipse:\;\rho \left(\theta \right)=\frac{ab}{\sqrt{a^{2}sin^{2}\left(\theta \right)+\;b^{2}cos^{2}\left(\theta \right)}}$$ (5)

$where\;\rho =radial\;coordinate;\;c=vertex\;curvature;\;K=conic\;constant;\;c_{bfs}=curvature\;of\;best\;fit\;sphere,\;\;u=\frac{\rho }{\rho_{max}},\;\;and\;M=2$. This value of M resulted in the best fit for the Forbes equation with the lowest slope variability [20].

Using the x-y coordinates, the coefficients for the Chien [19], Forbes [18,20,21], Fourier [2] and the elliptical [19] curves were calculated using MATLAB software (version 9.10, MathWorks Inc. Natick, MA, USA). To ensure the best Fourier curve fit, the Fourier coefficients for each lens were independently calculated instead of using the age-related coefficients given by Urs et al. [2].

In addition, Python software (version 3, Python Software Foundation) was used to evaluate different smoothing methods of the x-y raw data including 2^nd^ order Gaussian filtration [22–24] and wavelet smoothing [25,26]. The symlet 4 wavelet (S4W) method was smoothest and when fitted with 3^rd^ (3^rd^ Poly), 5^th^ (5^th^ Poly) and 7^th^ (7^th^ Poly) degree polynomials to maximize smoothness while minimizing the rmse of the fits, S4W 3^rd^ Poly was best.

### Curve parameters

Mean and standard deviation rmse fits to isolated lens sagittal profiles of the Chien, Forbes, Fourier, ellipse and S4W 3^rd^ Poly equations were compared. Curvature (k), waviness (W) [27–29] and curvature variance (kV) [30] of the curves for the central 6 mm diameter COZ were also assessed by applying the following equations:


$$\begin{array}{c} Curvature\;\left(\frac{1}{mm}\right)=\frac{\left|\frac{d^{2}y}{dx^{2}}\right|}{{\left(1+{\left(\frac{dy}{dx}\right)}^{2}\right)}^{\frac{3}{2}}} \end{array}$$ (6)

$$Waviness\;\left(\frac{1}{mm^{3}}\right)=\int_{0}^{s}{\left(\frac{dk}{ds}\right)}^{2}ds$$ (7)

$$Curvature\;Variance\;\left(\frac{1}{mm^{2}}\right)=\sum_{i=0}^{x}\frac{{\left(k_{i}-\bar{k}\right)}^{2}}{n-1}$$ (8)

where *s = *arc length, *k* = curvature, $\overset{―}{k}$ = mean curvature, and $k=\frac{1}{RoC}$

### Central optical power

Anterior and posterior RoC and COP were calculated for COZ diameters of 1 mm to 6 mm in 1 mm steps with the following formula [31]:


$$Central\;Optical\;Power\;\left(diopters\right)=\frac{n_{l}-n_{a}}{r_{a}}+\frac{n_{a}-n_{l}}{r_{p}}-\frac{t\left(n_{l}-n_{a}\right)\left(n_{a}-n_{l}\right)}{n_{l}r_{a}r_{p}}$$ (9)

where *n*_*a*_ = 1.336 and *n*_*l*_ = 1.42 are the indices of refraction of the aqueous humor/vitreous and lens, respectively; *r*_*a*_ and *r*_*p*_ (which is negative) are the anterior and posterior central lens surface RoCs, respectively; and *t* = central lens thickness.

**Fig 1:**
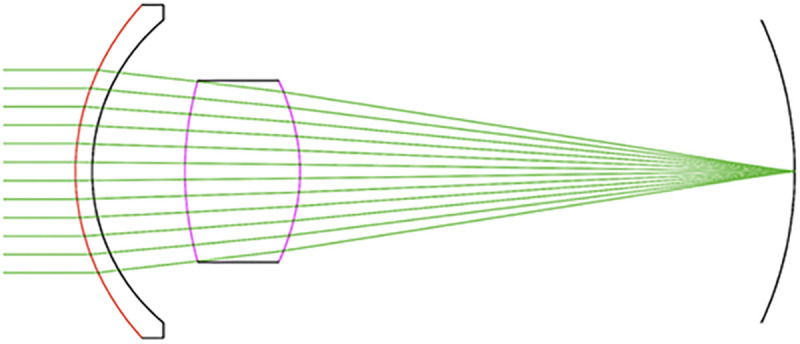
Optical software schematic of the Navarro eye model with the lens surfaces modified by the S4W 3^rd^ Poly equations.

The S4W 3^rd^ Poly fit was smoother than the S4W 5^th^ Poly and S4W 7^th^ Poly and had better fits, less waviness and lower curvature variances than the Chien, Forbes and ellipse equations. For these reasons, the S4W 3^rd^ Poly fits were imported into an optical software program (OpTaliX-Pro v 12.0, Optical Engineering Software) to form the lens surfaces of an eye model that incorporated corneal and anterior chamber parameters of the original Navarro eye model [32]. The RoCs of the lenses were assessed paraxially and with spherical arcs that had their vertices coincident with the lens vertex and their ends touching the lens surfaces at COZs of 1 mm, 2 mm 3 mm, 4 mm, 5 mm and 6 mm. Parallel rays of light were projected on the cornea of the model eye to evaluate RoCs and $Z_{4}^{0}$ as shown in Fig 1.

### Comparison of isolated lens and in vivo lens COP

To compare RoCs and COPs at the 3 mm COZ of the isolated lenses to *in vivo* lenses, the following formulae were used to account for the age-related growth of the lens [11,33,34]:


$$r_{av}=12.9mm-0.057\frac{mm}{year}\cdot a-0.61\frac{mm}{diopter}\cdot AA$$ (10)

$$r_{pv}=\mathrm{-6.2mm}+0.012\frac{mm}{year}\cdot a+0.13\frac{mm}{diopter}\cdot AA$$ (11)

$$t_{v}=2.93mm+0.024\frac{mm}{year}\cdot a+\left(5.8\cdot 10^{-2}\frac{mm}{diopter}-4.8\cdot 10^{-4}\frac{mm}{year\cdot diopter}\cdot a\right)\cdot AA$$ (12)

where *r*_*av*_ *and r*_*pv*_ *= in vivo* anterior and posterior lens RoCs, respectively, *t*_*v*_ = *in vivo* central lens thickness, *a* = age (years), and *AA *= accommodative amplitude (diopters).

## Results

### COP and spherical aberration

**Fig 2:**
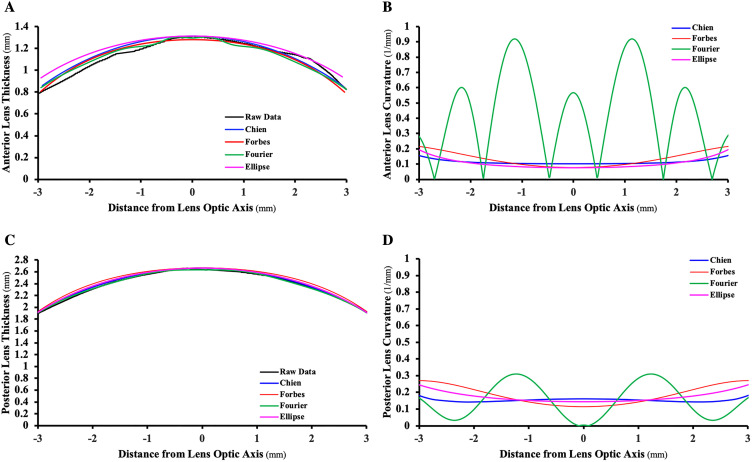
Chien, Forbes, Fourier and ellipse equations fits. Anterior lens A) thickness, B) curvature, and posterior lens C) thickness and D) curvature of the isolated lens from the 20 y/o donor.

Plots of the fits and curvature for the anterior and posterior surfaces of an isolated lens for the Chien, Forbes, Fourier and ellipse are given in Fig 2, which were similar to previous published results [35]. The Fourier has the most waviness and greatest curvature variation precluding it from reliably determining RoCs and consequently COP (see S2 Table).

**Fig 3:**
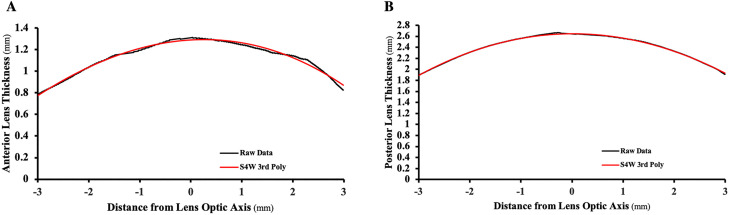
S4W 3^rd^ Poly equation fits. Anterior lens A) thickness and posterior lens B) thickness of the isolated lens from the 20 y/o donor.

The S4W 3^rd^ Poly fit the data significantly better and smoother than the other equations with a mean ± SD rmse fit = 10.9 ± 5.1 μm, waviness = 0.000011 ± 0.00008 1/mm^3^ and curvature variance = 0.0004 ± 0.0004 1/mm^2^ as shown in Table 1 and Fig 3.

**Table 1:** Means ± SD of isolated lens surface parameters.

Parameter	Chien	Forbes	Ellipse	S4W 3^rd^ Poly.
**Fit** (*μm*)	38.8 ± 18.7	63.2 ± 17.2	97.3 ± 30.2	10.9 ± 5.1
**W** ^a^ $\left(\frac{1}{mm^{3}}\right)$	0.020 ± 0.018	0.015 ± 0.022	0.032 ± 0.012	0.000011 ± 0.000008
**kV**^a^ ($\frac{1}{mm^{2}}$)	0.0006 ± 0.0006	0.0016 ± 0.0013	0.1510 ± 0.0938	0.0004 ± 0.0004
**ARoC**^b^ (*mm*)	10.8 ± 1.9	9.3 ± 1.6	11.4 ± 2.0	9.8 ± 1.4
**PRoC**^b^ (*mm*)	−6.5 ± 0.5	−6.0 ± 0.7	−8.1 ± 1.2	−5.8 ± 0.5
**t**^b^ (*mm*)	3.9 ± 0.2	3.9 ± 0.2	3.9 ± 0.2	3.9 ± 0.2

W = waviness; kV = curvature variance; ARoC = anterior radius of curvature;

PRoC = posterior radius of curvature; t = central thickness.

^a^6 mm diameter central optical zone.

^b^3 mm diameter central optical zone.

At the 3 mm COZ, the Chien, Forbes, elliptical and S4W 3^rd^ Poly curves all have anterior and posterior RoCs and COP that are consistent with an unaccommodated *in vivo* lens. The COP for the isolated lenses at the 3 mm COZ were 21.0 ± 1.9 diopters, 23.4 ± 2.7 diopters, 18.1 ± 2.3 and 23.1 ± 2.2 diopters for the Chien, Forbes, elliptical and S4W 3^rd^ Poly equations as given in Table 2, respectively. For the Forbes and elliptical equations, COP increased with distance from the optical zone, whilst for the Chien and S4W 3^rd^ Poly equations, it was the opposite; i.e., the COP decreased with distance from the optical zone.

**Table 2:** Means of all isolated lens central optical powers.

COZD (mm)	Chien (diopters)	Forbes (diopters)	Ellipse (diopters)	S4W 3^rd^Poly. (diopters)
**1**	23.4 ± 2.8	17.8 ± 2.4	16.1 ± 1.9	23.4 ± 2.2
**2**	22.3 ± 2.4	20.0 ± 2.3	16.9 ± 2.0	23.2 ± 2.2
**3**	21.0 ± 1.9	23.4 ± 2.7	18.1 ± 2.3	23.1 ± 2.2
**4**	19.2 ± 5.2	27.3 ± 3.6	20.3 ± 2.6	22.8 ± 2.2
**5**	17.8 ± 3.2	31.2 ± 4.6	23.9 ± 3.2	22.5 ± 2.2
**6**	17.9 ± 5.2	33.9 ± 5.0	29.9 ± 4.5	22.1 ± 2.2

COZD = central optical zone diameter.

Lens donors aged 20–30 years have lower limit accommodative amplitude range of 9.7 to 7.1 diopters, respectively [36]. If their isolated lenses were in the maximumly accommodated state, the mean COP (Eqs. 9–12) at a 3 mm diameter COZ should be 30.0 ± 1.0 diopters with a minimum ≥ 28.7 diopters [11,33,34]. At the 3 mm COZ the mean COP of the Chien, Forbes, elliptical and S4W 3^rd^ Poly equations was 21.4 diopters, which was essentially the same as the *in vivo* COP of 21.3 diopters as given in Table 3. This is consistent with a recent study that found in 20,004 cataract surgery patients an overall median intraocular lens power = 21.5 diopters [37]. Therefore, the isolated lenses had low COP consistent with the unaccommodated state.

**Table 3:** *In vivo* lens compared to isolated lens at 3 mm diameter central optical zone.

*In Vivo*										*In Vitro* *Isolated Lens*				
Age (*years*)	AA^*^ (*diopters*)	Accommodated				Unaccommodated								
		*r*_*av*_ (*mm*)	*r*_*pv*_ (*mm*)	*t*_*v*_ (*mm*)	*COP*_*v*_ (*diopters*)	*r*_*av*_ (*mm*)	*r*_*pv*_ (*mm*)	*t*_*v*_ (*mm*)	*COP*_*v*_ (*diopters*)	*COP*_*C*_ (*diopters*)	*COP*_*F*_ *(diopters)*	*COP*_*E*_ *(diopters)*	*COP*_*S4W*_ *(diopters)*	*Mean of all 4 Equations*
**20**	9.7	5.8	−4.7	3.9	31.6	11.8	−6.0	3.4	21.0	21.2	25.6	21.2	22.1	22.5
**21**	9.4	6.0	−4.7	3.9	31.2	11.7	−6.0	3.4	21.1	22.8	26.1	19.5	26.6	23.8
**22**	9.2	6.0	−4.7	3.9	31.0	11.7	−5.9	3.5	21.1	21.9	22.0	16.9	21.9	20.7
**24**	8.7	6.2	−4.8	3.9	30.4	11.5	−5.9	3.5	21.2	23.9	27.1	20.7	25.9	24.4
**25**	8.4	6.4	−4.8	3.9	30.1	11.5	−5.9	3.5	21.3	19.4	21.5	16.2	24.2	20.3
**26**	8.2	6.4	−4.8	3.9	29.9	11.4	−6.0	3.6	21.4	22.0	25.7	20.2	24.6	23.1
**27**	7.9	6.6	−4.9	3.9	29.5	11.4	−6.0	3.6	21.4	16.9	18.6	14.7	19.9	17.5
**28**	7.6	6.7	−4.9	3.9	29.2	11.3	−6.0	3.6	21.5	20.4	22.3	16.8	23.6	20.8
**29**	7.3	6.8	−4.9	4.0	28.9	11.3	−6.0	3.6	21.5	20.7	22.7	18.9	21.8	21.0
**30**	7.1	6.9	−4.9	4.0	28.7	11.2	−5.8	3.7	21.6	20.8	22.1	15.6	20.7	19.8
**Mean**	**8.4**	**6.4**	**−4.8**	**3.9**	**30.0**	**11.5**	**−5.90**	**3.53**	**21.3**	**21.0**	**23.4**	**18.1**	**23.1**	**21.4**
**SD**	0.9	0.4	0.1	0.02	1.0	0.2	0.04	0.1	0.2	1.9	2.7	2.3	2.2	2.1

AA^* ^= lower limit of accommodative amplitude; *r*_*a*_*, r*_*av*_*, t*_*v*_*, COP*_*v*_*,= in vivo* anterior, posterior radii of curvatures, central thickness and optical power, respectively; *COP*_*c,*_ *COP*_*F*_*, COP*_*E*_ *and COP*_*S4W*_* = *COP calculated using Chien, Forbes, ellipse, and S4W 3^rd^ Poly equations, respectively.

**Fig 4:**
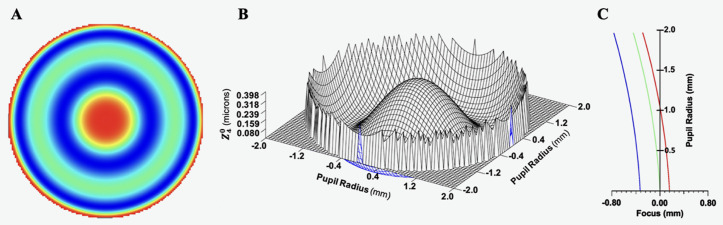
Graphs of spherical aberration for a 4 mm pupil of the model eye with the S4W 3^rd^ Poly equations fit to the surfaces of the isolated lens from the 20 y/o donor. **A)** $Z_{4}^{0}$ interferogram, **B)** $Z_{4}^{0}$ phase deformation and C) longitudinal chromatic aberration (wavelengths: blue = 450 nm, green = 550 nm and red = 650 nm).

From the optical software analysis of the eye model with S4W 3rd Poly lens surfaces, the spherical aberration coefficients $Z_{4}^{0}$ at a 4 mm diameter pupil were positive for all eyes with a mean of 0.2 ± 0.1 μm. The spherical aberration interferogram, $Z_{4}^{0}\;$ phase deformation, and longitudinal chromatic aberration are shown in Fig 4.

## Discussion

Independent of the equation used to model the isolated lenses, at the functional 3 mm diameter COZ, fresh *in vitro* isolated lenses from donors aged 20–30 years have the same range of low COP as do aged matched *in vivo* unaccommodated lenses. In addition, the spherical aberration of the eyes modelled with all ten isolated lenses had positive $Z_{4}^{0}$ coefficients consistent with *in vivo* unaccommodated eyes [38–41].

These findings align with finite element and mathematical analyses of intact lenses when the elastic modulus of the lens nucleus is specified as equal to or greater than the lens cortex as observed *in vivo* [42–45]. Furthermore, a finite element analysis using these lens nuclear moduli demonstrated that the total zonular force required to induce 10 diopters of accommodation is approximately 0.02 N [45], significantly less than the maximum force the ciliary muscle can apply of < 0.05 N [45]. Additionally, the same analysis indicated the total zonular force to transition the isolated lens to the unaccommodated state required 9.375 x 10^−5^ N, which falls within one standard deviation of the calculated mean total largest zonular force (8.66 x 10^−5^ ± 3.20 x 10^−5^ N) during the *in vivo* unaccommodated state of normal eyes with axial lengths of 22.0 to 24.5 mm [46].

Moreover, force diagram and balloon zonular force analyses of human and monkey lens capsules void of lens stroma [47,48] further support the present findings. These analyses demonstrated that during ciliary muscle contraction, equatorial zonular tension increases while simultaneously tension on the anterior and posterior zonules decreases as proposed by Schachar [45,47,48].

The increase in equatorial zonular tension during accommodation causes the observed increase in central lens thickness and COP that is associated with flattening of the peripheral lens surface [11], the universal negative shift in spherical aberration [39–41,49–54], stress on the lens capsule [55], movement of the lens equator towards the sclera [56,57], the lack of significant effect of gravity on accommodative amplitude [58], lens stability with head movements [59], and increased intra-lenticular hydrostatic pressure [60].

The present study relied on profilometer measurements of the lens surface, which inherently were not smooth. It is highly unlikely this significantly affected the results because at the 3 mm diameter COZ, independent of the wide range of the rmse fits of the equations, COP was low. Moreover, the findings are also compatible with the optical measurements reported by Helmholtz [4], Stradfelt [5], and Schachar [6].

**Fig 5:**
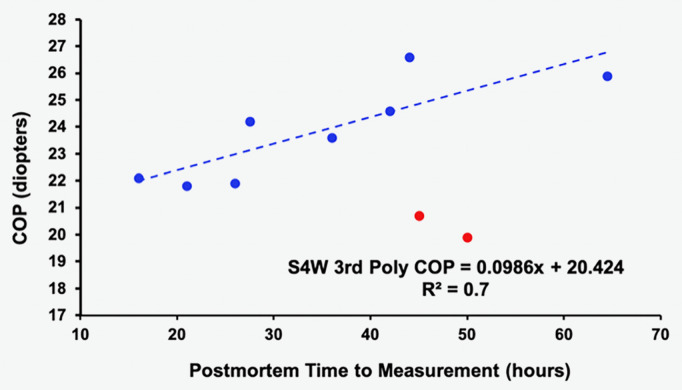
Linear regression analysis of the S4W 3^rd^ Poly fit COP versus postmortem time to measurement of the isolated lenses with R^2^ = 0.7 when two outliers are omitted (red dots).

The mean and median central thickness of the isolated lenses were 3.9 mm and 3.8 mm, respectively. Many *in vivo* studies for this age group using various techniques including Scheimpflug photography (3.53 mm [11], 3.7 mm [61]), A-scan ultrasound with velocity corrected for each subject (3.8 mm [62]), B-scan ultrasound (3.8 mm [63]), optical coherence tomography (3.75 mm [64]), and MRI (3.8 mm [65]) are similar. This implies that if there was any swelling of these fresh isolated lenses, it was minor, especially since swelling of the lenses would steepen their central surfaces, not flatten them [6]. This is consistent with a regression analysis of the S4W 3^rd^ Poly fit COP at 3 mm COZ vs. postmortem time to measurement of the isolated lenses in the present study. COP increased linearly with an R^2^ = 0.7 when two outliers are omitted as shown in Fig 5.

Although this study only involved a limited number of lenses, an isolated lens can only either be accommodated or unaccommodated. In this study, all the lenses had low COP as expected with the unaccommodated state. Given that the isolated lenses were fresh and COP was measured at COZ < 4 mm, a large number of lenses is unnecessary. In addition, the companion study that objectively and automatically measured the vertex RoCs of 12 fresh, young, isolated human lenses using interferometry also found that isolated lenses from donors aged 20–30 years had minimum COP consistent with unaccommodated lenses *in vivo* [66].

In conclusion, the isolated lens without zonular tension has low COP compatible with the *in vivo* unaccommodated state. Therefore relaxation of all the zonules cannot be the basis for the accommodative changes in lens shape.

## Supporting information

S1 Table This is the x-y coordinate data. (XLSX)

S2 Table This is the calculated parameter tables. (DOCX)
