# Supplementary material for: Central optical power of the isolated human lens without zonular tension
Source: PLoS One. 2025 Jul 1;20(7):e0326954. doi: 10.1371/journal.pone.0326954 (PMC12212553; doi:10.1371/journal.pone.0326954)
Supplement: S2 Table — (DOCX) [file pone.0326954.s002.docx]

**Lens Parameters and Central Optical Powers of the**

**Isolated Human Lenses Without Zonular Tension**

**Tables S1 to S4**

| **Table S1. Isolated lens curve parameters and central optical powers** | | | | | | | | | | | | | | | |
| --- | --- | --- | --- | --- | --- | --- | --- | --- | --- | --- | --- | --- | --- | --- | --- |
|  | | **20 y/o** | | | |  | **21 y/o** | | | |  | **22 y/o** | | | |
|  |  | **Chien** | **Forbes** | **Fourier** | **Ellipse** |  | **Chien** | **Forbes** | **Fourier** | **Ellipse** |  | **Chien** | **Forbes** | **Fourier** | **Ellipse** |
| **Fit** (μm) | **A** | 46.00 | 41.03 | 27.48 | 72.70 |  | 26.09 | 64.47 | 8.65 | 75.00 |  | 38.53 | 51.19 | 30.58 | 43.22 |
|  | **P** | 25.14 | 56.73 | 11.62 | 33.13 |  | 17.60 | 44.52 | 11.54 | 167.67 |  | 44.90 | 55.53 | 28.45 | 156.99 |
| **AL** (mm) | **A** | 9.09 | 9.08 | 9.08 | 9.21 |  | 10.05 | 9.65 | 10.04 | 10.06 |  | 10.71 | 9.73 | 10.70 | 10.82 |
|  | **P** | 11.22 | 10.11 | 11.33 | 11.10 |  | 10.23 | 9.90 | 10.23 | 10.49 |  | 10.23 | 9.66 | 10.05 | 10.69 |
| **E** $\left( \frac{1}{mm} \right)$ | **A** | 0.024 | 0.145 | 0.415 | 0.023 |  | 0.021 | 0.184 | 0.137 | 0.027 |  | 0.023 | 0.124 | 0.071 | 0.019 |
|  | **P** | 0.048 | 0.349 | 0.051 | 0.072 |  | 0.045 | 0.255 | 0.132 | 0.040 |  | 0.040 | 0.168 | 0.299 | 0.022 |
| **W** $\left( \frac{1}{{mm}^{3}} \right)$ | **A** | 0.016 | 0.015 | 48.437 | 0.060 |  | 0.007 | 0.019 | 14.328 | 0.045 |  | 0.001 | 0.002 | 0.090 | 0.120 |
|  | **P** | 0.019 | 0.020 | 1.724 | 0.039 |  | 0.078 | 0.000 | 11.326 | 0.046 |  | 0.013 | 0.000 | 30.510 | 0.029 |
| **kV** $\left( \frac{1}{{mm}^{2}} \right)$ | **A** | 0.0002 | 0.0020 | 0.0863 | 0.3661 |  | 0.0002 | 0.0027 | 0.0973 | 0.1480 |  | 0.0007 | 0.0022 | 0.0902 | 0.1200 |
|  | **P** | 0.0001 | 0.0030 | 0.0098 | 0.0214 |  | 0.0028 | 0.0000 | 0.0293 | 0.0686 |  | 0.0004 | 0.0000 | 0.0575 | 0.1161 |
| **t** (mm) |  | 4.0 | 4.0 | 4.0 | 4.0 |  | 3.8 | 3.8 | 3.8 | 3.8 |  | 3.8 | 3.8 | 3.8 | 3.8 |
| **RoC 1** (mm) | **A** | 9.9 | 12.1 | 14.8 | 13.0 |  | 7.8 | 11.6 | 164.6 | 10.9 |  | 10.9 | 14.1 | 3.6 | 11.4 |
|  | **P** | -6.3 | -8.5 | -9.0 | -6.9 |  | -4.8 | -5.7 | -7.7 | -9.0 |  | -5.8 | -6.7 | -15.8 | -11.1 |
| **RoC 2** (mm) | **A** | 9.8 | 10.2 | 1.2 | 12.3 |  | 8.2 | 9.5 | 2.0 | 10.4 |  | 10.3 | 11.5 | 57.1 | 10.9 |
|  | **P** | -6.5 | -7.0 | -3.5 | -6.6 |  | -5.3 | -5.7 | -2.0 | -8.6 |  | -6.1 | -6.7 | -1.6 | -10.6 |
| **RoC 3** (mm) | **A** | 9.5 | 8.1 | 1.8 | 11.0 |  | 8.9 | 7.4 | 2.2 | 9.5 |  | 9.4 | 8.9 | 5.7 | 10.1 |
|  | **P** | -6.8 | -5.5 | -3.6 | -6.2 |  | -6.3 | -5.7 | -2.3 | -7.9 |  | -6.5 | -6.7 | -1.4 | -9.8 |
| **RoC 4** (mm) | **A** | 9.0 | 6.5 | 2.1 | 9.4 |  | 9.9 | 5.8 | 34.1 | 8.3 |  | 8.2 | 6.9 | 18.8 | 9.0 |
|  | **P** | -7.0 | -4.5 | -10.2 | -5.6 |  | -8.0 | -5.7 | -25.5 | -7.0 |  | -7.1 | -6.7 | -33.7 | -8.7 |
| **RoC 5** (mm) | **A** | 8.1 | 5.3 | 3.4 | 7.4 |  | 10.9 | 4.8 | 12.1 | 6.8 |  | 7.0 | 5.5 | 4.4 | 7.7 |
|  | **P** | -6.8 | -4.0 | -23.3 | -4.9 |  | -11.3 | -5.8 | -13.3 | -5.9 |  | -7.9 | -6.8 | -3.0 | -7.4 |
| **RoC 6** (mm) | **A** | 6.5 | 4.6 | 3.5 | 5.2 |  | 10.5 | 4.2 | 4.5 | 5.2 |  | 5.7 | 4.7 | 3.4 | 6.2 |
|  | **P** | -5.5 | -3.8 | -6.0 | -4.1 |  | -18.4 | -6.0 | -3.0 | -4.6 |  | -8.9 | -7.0 | -6.2 | -5.9 |
| **Central optical zone diameter** (mm) | | | | | | | | | | | | | | | |
| **COP** (diopters) | **1** | 21.9 | 11.9 | 15.0 | 18.7 |  | 28.3 | 21.9 | 11.4 | 17.1 |  | 22.2 | 18.6 | 28.6 | 15.0 |
|  | **2** | 21.6 | 20.3 | 96.6 | 19.6 |  | 26.1 | 23.6 | 84.9 | 17.9 |  | 22.0 | 19.9 | 55.3 | 15.6 |
|  | **3** | 21.3 | 25.6 | 70.1 | 21.2 |  | 22.9 | 26.2 | 74.7 | 19.5 |  | 21.9 | 22.1 | 75.3 | 16.9 |
|  | **4** | 21.3 | 31.6 | 49.2 | 23.9 |  | 19.0 | 29.3 | 5.8 | 22.1 |  | 22.0 | 24.7 | 7.0 | 19.0 |
|  | **5** | 22.8 | 36.9 | 28.3 | 28.5 |  | 15.1 | 32.0 | 13.3 | 26.5 |  | 22.7 | 27.5 | 46.7 | 22.3 |
|  | **6** | 28.2 | 40.0 | 37.8 | 36.8 |  | 12.5 | 33.7 | 47.0 | 34.2 |  | 24.2 | 29.8 | 38.2 | 27.9 |

AL = arc length; E = bending energy; W = waviness; kV = curvature variance; t = central thickness; RoC = radius of curvature; A = anterior surface;

P = posterior surface; COP = central optical power

| **Table S2. Isolated lens curve parameters and central optical powers** | | | | | | | | | | | | | | | |  |  |  |  |  |  |  |  |  |  |  |  |  |  |  |
| --- | --- | --- | --- | --- | --- | --- | --- | --- | --- | --- | --- | --- | --- | --- | --- | --- | --- | --- | --- | --- | --- | --- | --- | --- | --- | --- | --- | --- | --- | --- |
|  | | **24 y/o** | | | |  | **25 y/o** | | | |  | **26 y/o** | | | |  |  |  |  |  |  |  |  |  |  |  |  |  |  |  |
|  |  | **Chien** | **Forbes** | **Fourier** | **Ellipse** |  | **Chien** | **Forbes** | **Fourier** | **Ellipse** |  | **Chien** | **Forbes** | **Fourier** | **Ellipse** |  |  |  |  |  |  |  |  |  |  |  |  |  |  |  |
| **Fit** (μm) | **A** | 20.96 | 37.73 | 17.33 | 73.06 |  | 51.34 | 65.91 | 21.05 | 74.61 |  | 46.96 | 78.36 | 21.79 | 52.11 |  |  |  |  |  |  |  |  |  |  |  |  |  |  |  |
|  | **P** | 16.98 | 55.12 | 14.17 | 101.48 |  | 32.14 | 79.18 | 19.48 | 159.57 |  | 33.94 | 60.10 | 23.99 | 128.76 |  |  |  |  |  |  |  |  |  |  |  |  |  |  |  |
| **AL** (mm) | **A** | 9.90 | 9.52 | 9.82 | 9.93 |  | 10.52 | 10.20 | 10.51 | 10.49 |  | 10.35 | 9.96 | 10.34 | 10.30 |  |  |  |  |  |  |  |  |  |  |  |  |  |  |  |
|  | **P** | 10.97 | 10.57 | 11.05 | 11.21 |  | 11.01 | 10.60 | 11.00 | 11.21 |  | 10.96 | 10.60 | 10.97 | 11.18 |  |  |  |  |  |  |  |  |  |  |  |  |  |  |  |
| **E** $\left( \frac{1}{mm} \right)$ | **A** | 0.018 | 0.139 | 0.060 | 0.021 |  | 0.011 | 0.107 | 0.073 | 0.013 |  | 0.019 | 0.174 | 0.047 | 0.022 |  |  |  |  |  |  |  |  |  |  |  |  |  |  |  |
|  | **P** | 0.065 | 0.387 | 0.098 | 0.058 |  | 0.032 | 0.185 | 0.121 | 0.025 |  | 0.048 | 0.314 | 0.060 | 0.045 |  |  |  |  |  |  |  |  |  |  |  |  |  |  |  |
| **W** $\left( \frac{1}{{mm}^{3}} \right)$ | **A** | 0.017 | 0.009 | 3.456 | 0.040 |  | 0.001 | 0.017 | 8.580 | 0.022 |  | 0.016 | 0.037 | 0.377 | 0.035 |  |  |  |  |  |  |  |  |  |  |  |  |  |  |  |
|  | **P** | 0.006 | 0.011 | 5.693 | 0.036 |  | 0.060 | 0.001 | 9.561 | 0.026 |  | 0.043 | 0.004 | 1.831 | 0.036 |  |  |  |  |  |  |  |  |  |  |  |  |  |  |  |
| **kV** $\left( \frac{1}{{mm}^{2}} \right)$ | **A** | 0.0006 | 0.0012 | 0.0763 | 0.2115 |  | 0.0000 | 0.0022 | 0.0904 | 0.2380 |  | 0.0002 | 0.0051 | 0.0631 | 0.1484 |  |  |  |  |  |  |  |  |  |  |  |  |  |  |  |
|  | **P** | 0.0002 | 0.0016 | 0.0237 | 0.0257 |  | 0.0019 | 0.0002 | 0.0327 | 0.0704 |  | 0.0016 | 0.0006 | 0.0024 | 0.0357 |  |  |  |  |  |  |  |  |  |  |  |  |  |  |  |
| **t** (mm) |  | 4.2 | 4.2 | 4.2 | 4.2 |  | 3.7 | 3.7 | 3.7 | 3.7 |  | 4.1 | 4.1 | 4.1 | 4.1 |  |  |  |  |  |  |  |  |  |  |  |  |  |  |  |
| **RoC 1** (mm) | **A** | 7.6 | 10.5 | 5.8 | 12.1 |  | 11.0 | 15.5 | 18.7 | 13.8 |  | 10.6 | 17.7 | 4.8 | 11.3 |  |  |  |  |  |  |  |  |  |  |  |  |  |  |  |
|  | **P** | -5.2 | -7.0 | -16.4 | -7.3 |  | -6.0 | -7.0 | -2.3 | -9.7 |  | -4.9 | -6.6 | -6.9 | -8.0 |  |  |  |  |  |  |  |  |  |  |  |  |  |  |  |
| **RoC 2** (mm) | **A** | 8.2 | 9.3 | 4.8 | 11.5 |  | 11.3 | 12.5 | 2.9 | 13.2 |  | 10.5 | 12.4 | 32.0 | 10.8 |  |  |  |  |  |  |  |  |  |  |  |  |  |  |  |
|  | **P** | -5.4 | -6.0 | -2.6 | -7.1 |  | -6.0 | -7.0 | -2.3 | -9.7 |  | -5.3 | -6.0 | -4.2 | -7.7 |  |  |  |  |  |  |  |  |  |  |  |  |  |  |  |
| **RoC 3** (mm) | **A** | 9.2 | 7.9 | 117.9 | 10.5 |  | 11.6 | 9.6 | 2.9 | 12.2 |  | 10.2 | 8.3 | 28.4 | 9.9 |  |  |  |  |  |  |  |  |  |  |  |  |  |  |  |
|  | **P** | -5.7 | -5.1 | -2.4 | -6.6 |  | -6.9 | -6.6 | -1.9 | -9.0 |  | -6.1 | -5.4 | -4.0 | -7.2 |  |  |  |  |  |  |  |  |  |  |  |  |  |  |  |
| **RoC 4** (mm) | **A** | 10.9 | 6.7 | 11.6 | 9.1 |  | 12.0 | 7.4 | 31.3 | 10.8 |  | 9.7 | 5.9 | 10.1 | 8.8 |  |  |  |  |  |  |  |  |  |  |  |  |  |  |  |
|  | **P** | -6.1 | -4.5 | -7.1 | -6.0 |  | -8.5 | -6.2 | -6.2 | -8.1 |  | -7.3 | -4.9 | -5.4 | -6.5 |  |  |  |  |  |  |  |  |  |  |  |  |  |  |  |
| **RoC 5** (mm) | **A** | 13.7 | 5.7 | 5.0 | 7.5 |  | 12.1 | 5.8 | 1722.6 | 9.1 |  | 8.6 | 4.5 | 3.9 | 7.3 |  |  |  |  |  |  |  |  |  |  |  |  |  |  |  |
|  | **P** | -6.4 | -4.1 | -137.8 | -5.2 |  | -11.6 | -5.9 | -9.5 | -7.0 |  | -9.1 | -4.7 | -6.6 | -5.7 |  |  |  |  |  |  |  |  |  |  |  |  |  |  |  |
| **RoC 6** (mm) | **A** | 16.5 | 5.1 | 2.4 | 5.7 |  | 11.2 | 4.9 | 3.7 | 7.2 |  | 6.9 | 3.8 | 6.0 | 5.7 |  |  |  |  |  |  |  |  |  |  |  |  |  |  |  |
|  | **P** | -6.2 | -4.2 | -4.6 | -4.3 |  | -18.3 | -5.8 | -8.7 | -5.7 |  | -11.0 | -4.7 | -5.3 | -4.7 |  |  |  |  |  |  |  |  |  |  |  |  |  |  |  |
| **Central optical zone diameter** (mm) | | | | | | | | | | | | | | | |  | 27.1 | 20.4 | 19.7 | 18.4 |  | 23.0 | 17.1 | 8.1 | 14.4 |  | 34.1 | 25.8 | 24.4 | 20.9 |
| **COP** (diopters) | **1** | 27.1 | 20.1 | 19.7 | 18.4 |  | 21.7 | 17.5 | 41.5 | 14.8 |  | 25.0 | 17.5 | 29.6 | 17.9 |  |  |  |  |  |  |  |  |  |  |  |  |  |  |  |
|  | **2** | 25.8 | 22.9 | 49.7 | 19.2 |  | 21.6 | 18.8 | 66.4 | 15.1 |  | 23.8 | 20.7 | 22.7 | 18.6 |  |  |  |  |  |  |  |  |  |  |  |  |  |  |  |
|  | **3** | 23.8 | 27.0 | 36.4 | 20.7 |  | 19.4 | 21.6 | 72.6 | 16.2 |  | 22.0 | 25.6 | 24.2 | 20.1 |  |  |  |  |  |  |  |  |  |  |  |  |  |  |  |
|  | **4** | 21.5 | 31.5 | 19.1 | 23.2 |  | 16.8 | 25.0 | 16.3 | 18.1 |  | 20.2 | 31.3 | 23.8 | 22.5 |  |  |  |  |  |  |  |  |  |  |  |  |  |  |  |
|  | **5** | 19.3 | 35.0 | 17.5 | 27.3 |  | 14.2 | 28.6 | 8.9 | 21.2 |  | 19.0 | 36.7 | 34.1 | 26.3 |  |  |  |  |  |  |  |  |  |  |  |  |  |  |  |
|  | **6** | 18.6 | 36.6 | 52.8 | 34.2 |  | 12.1 | 31.7 | 32.2 | 26.3 |  | 19.9 | 40.1 | 29.8 | 32.8 |  |  |  |  |  |  |  |  |  |  |  |  |  |  |  |

AL = arc length; E = bending energy; W = waviness; kV = curvature variance; t = central thickness; RoC = radius of curvature; A = anterior surface;

P = posterior surface; COP = central optical power

| **Table S3. Isolated lens curve parameters and central optical powers** | | | | | | | | | | | | | | | |  |  |  |  |  |  |  |  |  |  |  |  |  |  |  |
| --- | --- | --- | --- | --- | --- | --- | --- | --- | --- | --- | --- | --- | --- | --- | --- | --- | --- | --- | --- | --- | --- | --- | --- | --- | --- | --- | --- | --- | --- | --- |
|  | | **27 y/o** | | | |  | **28 y/o** | | | |  | **29 y/o** | | | |  |  |  |  |  |  |  |  |  |  |  |  |  |  |  |
|  |  | **Chien** | **Forbes** | **Fourier** | **Ellipse** |  | **Chien** | **Forbes** | **Fourier** | **Ellipse** |  | **Chien** | **Forbes** | **Fourier** | **Ellipse** |  |  |  |  |  |  |  |  |  |  |  |  |  |  |  |
| **Fit** (μm) | **A** | 73.35 | 89.08 | 12.30 | 80.27 |  | 46.31 | 71.06 | 23.38 | 67.92 |  | 25.82 | 84.75 | 11.50 | 43.83 |  |  |  |  |  |  |  |  |  |  |  |  |  |  |  |
|  | **P** | 39.66 | 75.07 | 26.05 | 132.92 |  | 18.56 | 46.63 | 16.83 | 156.73 |  | 35.42 | 72.52 | 22.23 | 107.28 |  |  |  |  |  |  |  |  |  |  |  |  |  |  |  |
| **AL** (mm) | **A** | 10.87 | 10.33 | 10.80 | 10.72 |  | 10.35 | 9.96 | 10.29 | 10.32 |  | 10.82 | 10.21 | 10.80 | 10.70 |  |  |  |  |  |  |  |  |  |  |  |  |  |  |  |
|  | **P** | 11.32 | 10.90 | 11.35 | 11.51 |  | 10.89 | 10.63 | 10.94 | 11.23 |  | 10.76 | 10.47 | 10.78 | 10.97 |  |  |  |  |  |  |  |  |  |  |  |  |  |  |  |
| **E** $\left( \frac{1}{mm} \right)$ | **A** | 0.006 | 0.064 | 0.084 | 0.009 |  | 0.011 | 0.092 | 0.079 | 0.012 |  | 0.017 | 0.163 | 0.024 | 0.024 |  |  |  |  |  |  |  |  |  |  |  |  |  |  |  |
|  | **P** | 0.026 | 0.153 | 0.219 | 0.020 |  | 0.042 | 0.231 | 0.236 | 0.030 |  | 0.035 | 0.222 | 0.038 | 0.031 |  |  |  |  |  |  |  |  |  |  |  |  |  |  |  |
| **W** $\left( \frac{1}{{mm}^{3}} \right)$ | **A** | 0.001 | 0.012 | 8.100 | 0.014 |  | 0.002 | 0.016 | 11.170 | 0.022 |  | 0.004 | 0.035 | 0.133 | 0.031 |  |  |  |  |  |  |  |  |  |  |  |  |  |  |  |
|  | **P** | 0.019 | 0.003 | 20.551 | 0.020 |  | 0.030 | 0.000 | 22.259 | 0.029 |  | 0.010 | 0.011 | 0.323 | 0.032 |  |  |  |  |  |  |  |  |  |  |  |  |  |  |  |
| **kV** $\left( \frac{1}{{mm}^{2}} \right)$ | **A** | 0.0000 | 0.0014 | 0.0868 | 0.3108 |  | 0.0000 | 0.0020 | 0.0951 | 0.2862 |  | 0.0001 | 0.0047 | 0.0628 | 0.1035 |  |  |  |  |  |  |  |  |  |  |  |  |  |  |  |
|  | **P** | 0.0006 | 0.0004 | 0.0415 | 0.0750 |  | 0.0010 | 0.0000 | 0.0469 | 0.0550 |  | 0.0004 | 0.0016 | 0.0016 | 0.0646 |  |  |  |  |  |  |  |  |  |  |  |  |  |  |  |
| **t** (mm) |  | 3.7 | 3.7 | 3.7 | 3.7 |  | 3.8 | 3.8 | 3.8 | 3.8 |  | 4.1 | 4.1 | 4.1 | 4.1 |  |  |  |  |  |  |  |  |  |  |  |  |  |  |  |
| **RoC 1** (mm) | **A** | 14.0 | 19.2 | 3.0 | 15.7 |  | 11.5 | 16.9 | 6.5 | 14.4 |  | 8.8 | 17.9 | 7.8 | 10.6 |  |  |  |  |  |  |  |  |  |  |  |  |  |  |  |
|  | **P** | -6.5 | -8.4 | -13.0 | -10.7 |  | -5.3 | -6.2 | -353.6 | -9.3 |  | -6.1 | -8.9 | -7.1 | -9.4 |  |  |  |  |  |  |  |  |  |  |  |  |  |  |  |
| **RoC 2** (mm) | **A** | 14.3 | 15.7 | 3.1 | 15.1 |  | 11.7 | 13.6 | 2.6 | 13.7 |  | 9.2 | 12.6 | 5.7 | 10.2 |  |  |  |  |  |  |  |  |  |  |  |  |  |  |  |
|  | **P** | -6.8 | -7.9 | -2.0 | -10.3 |  | -5.6 | -6.1 | -1.8 | -8.9 |  | -6.4 | -7.7 | -5.6 | -9.0 |  |  |  |  |  |  |  |  |  |  |  |  |  |  |  |
| **RoC 3** (mm) | **A** | 14.8 | 12.2 | 35.0 | 14.0 |  | 11.9 | 10.4 | 7.5 | 12.6 |  | 9.8 | 8.6 | 6.2 | 9.4 |  |  |  |  |  |  |  |  |  |  |  |  |  |  |  |
|  | **P** | -7.5 | -7.2 | -1.5 | -9.6 |  | -6.3 | -5.9 | -1.5 | -8.3 |  | -6.9 | -6.5 | -6.7 | -8.4 |  |  |  |  |  |  |  |  |  |  |  |  |  |  |  |
| **RoC 4** (mm) | **A** | 15.3 | 9.3 | 3.6 | 12.5 |  | 12.2 | 7.9 | 3.9 | 11.1 |  | 10.5 | 6.1 | 9.0 | 8.4 |  |  |  |  |  |  |  |  |  |  |  |  |  |  |  |
|  | **P** | -8.5 | -6.6 | -3.8 | -8.7 |  | -7.2 | -5.8 | -8.7 | -7.5 |  | -7.7 | -5.5 | -9.6 | -7.5 |  |  |  |  |  |  |  |  |  |  |  |  |  |  |  |
| **RoC 5** (mm) | **A** | 15.4 | 7.3 | 15.6 | 10.6 |  | 12.1 | 6.2 | 45.8 | 9.3 |  | 11.0 | 4.6 | 10.6 | 7.1 |  |  |  |  |  |  |  |  |  |  |  |  |  |  |  |
|  | **P** | -10.0 | -6.1 | -3.8 | -7.6 |  | -8.6 | -5.8 | -3.2 | -6.5 |  | -8.5 | -4.8 | -6.4 | -6.5 |  |  |  |  |  |  |  |  |  |  |  |  |  |  |  |
| **RoC 6** (mm) | **A** | 14.1 | 6.0 | 2.7 | 8.5 |  | 10.9 | 5.1 | 2.3 | 7.3 |  | 10.1 | 3.9 | 12.7 | 5.7 |  |  |  |  |  |  |  |  |  |  |  |  |  |  |  |
|  | **P** | -11.9 | -5.7 | -11.0 | -6.3 |  | -10.3 | -5.9 | -12.0 | -5.3 |  | -8.9 | -4.5 | -3.9 | -5.2 |  |  |  |  |  |  |  |  |  |  |  |  |  |  |  |
| **Central optical zone diameter** (mm) | | | | | | | | | | | | | | | |  | 27.1 | 20.4 | 19.7 | 18.4 |  | 23.0 | 17.1 | 8.1 | 14.4 |  | 34.1 | 25.8 | 24.4 | 20.9 |
| **COP** (diopters) | **1** | 19.0 | 14.4 | 34.5 | 13.2 |  | 23.2 | 18.5 | 13.2 | 14.9 |  | 23.2 | 14.2 | 22.6 | 16.8 |  |  |  |  |  |  |  |  |  |  |  |  |  |  |  |
|  | **2** | 18.2 | 16.0 | 69.0 | 13.7 |  | 22.1 | 20.0 | 80.1 | 15.6 |  | 22.2 | 17.6 | 29.7 | 17.5 |  |  |  |  |  |  |  |  |  |  |  |  |  |  |  |
|  | **3** | 16.9 | 18.6 | 60.0 | 14.7 |  | 20.4 | 22.3 | 67.6 | 16.8 |  | 20.7 | 22.8 | 26.1 | 18.9 |  |  |  |  |  |  |  |  |  |  |  |  |  |  |  |
|  | **4** | 15.4 | 21.8 | 45.1 | 16.3 |  | 18.5 | 25.1 | 31.5 | 18.7 |  | 19.0 | 29.2 | 18.1 | 21.2 |  |  |  |  |  |  |  |  |  |  |  |  |  |  |  |
|  | **5** | 13.9 | 25.3 | 27.2 | 18.9 |  | 16.8 | 28.0 | 27.7 | 21.9 |  | 17.6 | 35.7 | 21.1 | 24.8 |  |  |  |  |  |  |  |  |  |  |  |  |  |  |  |
|  | **6** | 13.0 | 28.7 | 38.3 | 23.1 |  | 15.9 | 30.5 | 43.2 | 27.3 |  | 17.7 | 40.4 | 28.0 | 30.9 |  |  |  |  |  |  |  |  |  |  |  |  |  |  |  |

AL = arc length; E = bending energy; W = waviness; kV = curvature variance; t = central thickness; RoC = radius of curvature; A = anterior surface;

P = posterior surface; COP = central optical power

| **Table S4. Isolated lens curve parameters and central optical powers** | | | | | | | | | | | | | |
| --- | --- | --- | --- | --- | --- | --- | --- | --- | --- | --- | --- | --- | --- |
| **30 y/o**        **30 y/o** | | | | | | |  |  |  |  |  |  |  |
|  | | **Chien** | **Forbes** | **Fourier** | **Ellipse** | | |  |  |  |  |  |  |
| **Fit** (μm) | **A** | 44.88 | 45.15 | 30.97 | 71.23 | | |  |  |  |  |  |  |
|  | **P** | 90.54 | 96.38 | 63.02 | 147.79 | | |  |  |  |  |  |  |
| **AL** (mm) | **A** | 10.05 | 9.75 | 9.91 | 10.19 | | |  |  |  |  |  |  |
|  | **P** | 11.50 | 10.95 | 11.56 | 11.63 | | |  |  |  |  |  |  |
| **E** $\left( \frac{1}{mm} \right)$ | **A** | 0.011 | 0.062 | 1.643 | 0.008 | | |  |  |  |  |  |  |
|  | **P** | 0.035 | 0.247 | 0.109 | 0.033 | | |  |  |  |  |  |  |
| **W** $\left( \frac{1}{{mm}^{3}} \right)$ | **A** | 0.005 | 0.004 | 213.114 | 0.016 | | |  |  |  |  |  |  |
|  | **P** | 0.029 | 0.001 | 10.163 | 0.025 | | |  |  |  |  |  |  |
| **kV** $\left( \frac{1}{{mm}^{2}} \right)$ | **A** | 0.0001 | 0.0004 | 0.2598 | 0.5188 | | |  |  |  |  |  |  |
|  | **P** | 0.0010 | 0.0002 | 0.0259 | 0.0372 | | |  |  |  |  |  |  |
| **t** (mm) |  | 3.8 | 3.8 | 3.8 | 3.8 | | |  |  |  |  |  |  |
| **RoC 1** (mm) | **A** | 13.0 | 13.7 | 2.2 | 17.3 | | |  |  |  |  |  |  |
|  | **P** | -5.6 | -6.6 | -16.4 | -8.7 | | |  |  |  |  |  |  |
| **RoC 2** (mm) | **A** | 12.8 | 12.6 | 0.8 | 16.5 | | |  |  |  |  |  |  |
|  | **P** | -6.0 | -6.2 | -2.3 | -8.3 | | |  |  |  |  |  |  |
| **RoC 3** (mm) | **A** | 12.3 | 11.1 | 0.7 | 15.2 | | |  |  |  |  |  |  |
|  | **P** | -6.7 | -5.8 | -2.2 | -7.8 | | |  |  |  |  |  |  |
| **RoC 4** (mm) | **A** | 11.7 | 9.6 | 2.8 | 13.4 | | |  |  |  |  |  |  |
|  | **P** | -7.9 | -5.5 | -16.2 | -7.1 | | |  |  |  |  |  |  |
| **RoC 5** (mm) | **A** | 10.7 | 8.4 | 1.0 | 11.2 | | |  |  |  |  |  |  |
|  | **P** | -9.5 | -5.3 | -5.7 | -6.3 | | |  |  |  |  |  |  |
| **RoC 6** (mm) | **A** | 9.2 | 7.3 | 5.2 | 8.7 | | |  |  |  |  |  |  |
|  | **P** | -11.2 | -5.3 | -8.0 | -5.3 | | |  |  |  |  |  |  |
| **Central optical zone diameter** (mm) | | | | | |  | | |  |  |  |  |  |
| **COP** (diopters) | **1** | 21.4 | 19.0 | 42.8 | 14.5 | | |  |  |  |  |  |  |
|  | **2** | 20.5 | 20.2 | 144.7 | 15.1 | | |  |  |  |  |  |  |
|  | **3** | 19.3 | 21.9 | 163.5 | 16.3 | | |  |  |  |  |  |  |
|  | **4** | 17.9 | 23.9 | 35.1 | 18.0 | | |  |  |  |  |  |  |
|  | **5** | 16.8 | 25.8 | 97.9 | 20.9 | | |  |  |  |  |  |  |
|  | **6** | 16.6 | 27.2 | 26.5 | 25.6 | | |  |  |  |  |  |  |

AL = arc length; E = bending energy; W = waviness;

kV = curvature variance; t = central thickness;

RoC = radius of curvature; A = anterior surface;

P = posterior surface; COP = central optical power
